# A regulatory domain spanning the repeat sequence RE1 from herpes simplex virus type 1 has cell specific differential functions in trigeminal neurons and fibroblasts

**DOI:** 10.1016/j.febslet.2009.09.037

**Published:** 2009-10-20

**Authors:** Hannah C. Stevens, Carolyn Fiskerstrand, Vivien J. Bubb, Robert Dalziel, John P. Quinn

**Affiliations:** a School of Biomedical Sciences, Divisions of Physiology and HACB, University of Liverpool, L69 3BX, UK; b The Roslin Institute and R(D)SVS, University of Edinburgh, Summerhall, Edinburgh EH9 1QH, UK

**Keywords:** VNTR, variable number tandem repeat, HSV-1, herpes simplex virus type 1, TAC1, preprotachykinin-A, TG, trigeminal ganglia, RE1, reiteration element 1, CTCF, CCCTC binding protein, DRG, dorsal root ganglia, CTRL1, CTCF tandem repeat long 1, CTRL2, CTCF tandem repeat long 2, SLC6A3, dopamine transporter gene, SLC6A4, serotonin transporter gene, BHK, baby hamster kidney, BGP-1, beta globin protein 1, Herpes simplex virus type 1, Cell specific enhancer, Tandem repeat, CCCTC binding protein

## Abstract

In this report we demonstrate that the herpes simplex virus type 1 reiteration element 1 (RE1) (nt: 117158–117353) in concert with its flanking sequences is both a cell specific and stimulus inducible regulatory domain. This region of the virus genome and specifically the RE1 supports differential reporter gene expression in both baby hamster kidney cells and disassociated rat trigeminal ganglia and is present within a region that is implicated in regulating latency of the virus in neuronal cells. Further we demonstrate that this locus is a transcriptional regulatory domain and a target for the transcription factor CCCTC binding protein.

## 1 Introduction

Repetitive DNA is a common feature of eukaryotic DNA and many functions have been ascribed to it including a role in transcriptional regulation and modulation of DNA structure [1]. We have shown that polymorphic repeats, termed variable number tandem repeats (VNTR), can act as cell specific and stimulus inducible enhancers in neuronal cells [1–4]. Herpes simplex virus type 1 (HSV-1) primarily infects the host via fibroblasts this is the site were the lytic phase can occur, while HSV-1 can establish and reactivate from latency in neurons. Therefore, regulation of the HSV-1 lifecycle requires cell specific enhancers. A role of tandem repeat DNA as cell specific elements is reflected in many viruses, including HSV-1, in which tandem repeats differentially regulate transcription [5].

**Fig. 1:**
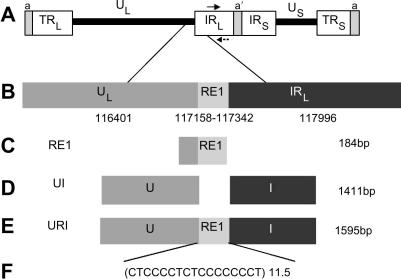
The loci studied in the context of the HSV-1 genome. There are a number of genes in this region running in both orientations. References to nucleotide position are from the HSV-1 genome sequence (NC_001806). (A) The structure of the HSV-1 genome showing proximity of the LAT (
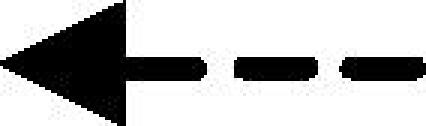
) and ICP0 (→) genes to the RE1 (IR_L_ boundary with the unique long (U_L_) region). The RE1 element is also present in the TR_L_ region (nt: 9018–9213). (B) The loci to be investigated illustrating the CT rich RE1 (184 bp) and GC rich U and I regions. (C) The RE1 was cloned into pGL3p and pGL3c (Promega) upstream of the SV40 promoter (Sac1/Xho sites) (nt: 116 996–117 360; 364 bp). It was not possible to isolate the RE1 without some flanking sequence due to the repetitive nature of the sequence. (D) The UI construct was cloned into pGL3p (1400 bp). (E) The URI construct was cloned into pGL3p (1595 bp). (F) The RE1 contains 11.5 copies of a CT rich repeat sequence.

The rat preprotachykinin-A (TAC1) gene which encodes the neuropeptide substance P, has an intronic repeat of CCCTCCC, which can support cell specific reporter gene expression in the context of a reporter gene construct [6]. The TAC1 gene is a stimulus inducible gene in trigeminal ganglia (TG) neurons [7], similarly reactivation of HSV-1 is stress inducible in TG [8]. Despite both the constraint on virus genome size, and the inherent instability of tandem repeats, repetitive regions within the HSV-1 genome are conserved, show copy number variation and are hot spots for recombination [9–11]. We have identified a repetitive domain termed reiteration element 1 (RE1) [12], also known as CCCTC binding protein (CTCF) repeat long 1 (CTRL1) [13]. This element has a CT repetitive structure similar to the TAC1 intronic repeat, Fig. 1A and has been shown to bind to CTCF in latently infected mouse dorsal root ganglia (DRG) using in vivo ChIP analysis [13].

In addition to the data on the TAC1 gene and HSV-1 RE1 we have more recently shown that tandem repeats within the human dopamine and serotonin transporter genes (SLC6A3 and SLC6A4, respectively) can support differential cell and stimulus inducible expression in vivo and in vitro [1–4]. One of the transcription factors that modulates expression of the SLC6A4 repeat domain is CTCF [3,4], which has a high affinity for the “CCCTC” element which is repeated in RE1 [14]. We also wanted to investigate the RE1 within the context of a larger locus, URI, which spans from 116 401 bp to 117 996 bp of the HSV-1 genome (Fig. 1). The URI region is located upstream of the latency associated transcript (LAT) promoter near the unique-long/internal repeat long (U_L_/IR_L_) junction. We therefore set out to address the hypothesis that the RE1, URI and UI, which is similar to URI but has the RE1 region removed (Fig. 1), can act as cell specific and stimulus inducible regulators of reporter gene expression which could be further modulated by CTCF.

## 2 Materials and methods

### 2.1 Generation of reporter gene constructs

The RE1, UI and URI regions were cloned upstream of the minimum SV40 promoter in the pGL3 promoter vector (Fig. 1). The CTCF expression construct consisted of CTCF cDNA cloned into the mammalian expression vector pcDNA3 (Invitrogen, San Diego, CA) and was a kind gift from E. Klenova, University of Essex.

### 2.2 Tissue culture and transfection

Baby hamster kidney (BHK) fibroblasts were transfected with Exgen 500 in vitro transfection reagent (Fermentas) using the standard protocol a transfection efficiency of 80–90% was achieved. BHK fibroblasts were maintained in Glasgow’s medium (Invitrogen) supplemented with 10% FCS and 1% (v/v) 100× penicillin/streptomycin (equates to a final concentration of 100 units penicillin/100 μg streptomycin).

Disassociated TG were prepared from Wistar rats (2–5 days old) and cultured based on a previously published protocol [15]. TG were transfected using lipofectamine and the standard protocol was followed (Invitrogen) an average transfection efficiency of 60% was achieved. In brief, 1 μg reporter gene and CTCF expression vector were transfected per 100 000 cells in 24 well plate format. A minimal TK promoter expressing *renillia* luciferase (pMLuc-2, 0.1 μg/well, Novagen) was added as an internal control in all experiments. Reporter gene expression was analyzed 48 h after transfection using the Dual Luciferase Reporter Assay System (Promega) and assayed for both luciferase and renillin activity using a luminometer (GLOMAX 96 microplate luminometer). The mean normalized luciferase values were calculated together with the SEM (luciferase/renillin). To normalise for transfection efficiency the total DNA concentration was standardized by co-transfection of reporter constructs with 1 μg of CTCF or 1 μg of pGL3b (Promega). The CTCF data was normalised to transgene expression from pGL3p co-transfected with CTCF.

## 3 Results and discussion

**Fig. S1:**
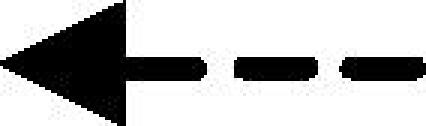
Primary neuronal cultures with neuronal specific antibody shows that the cultures are approximately 70% neuronal Images are composites with the DAPI shown blue and the Alexa Fluor 488 shown green (Bar 10μM). (A) Control Secondary antibody (Mouse IgG conjugated to Alexa Fluor 488) only (B) Field of neuronal cells with a neuronal specific (Neu-N) antibody (C and D).

**Fig. 2:**
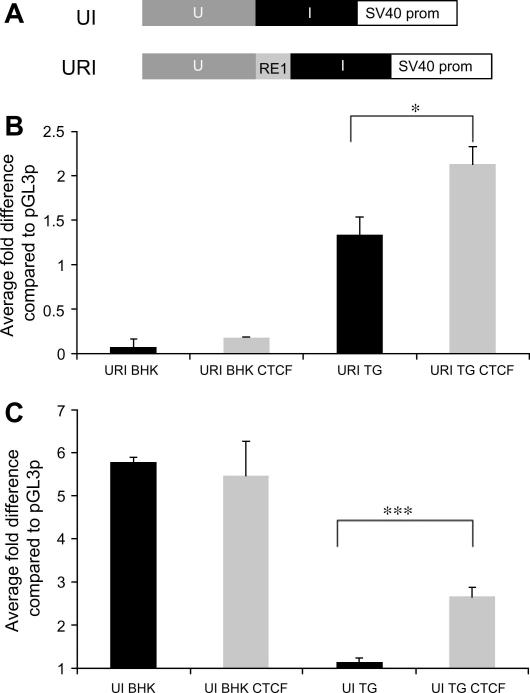
UI and URI are cell specific domains; CTCF significantly activates transcriptional activity of the UI and URI elements in TG. (A) The structure of the inserts UI and URI in pGL3p. (B) The URI construct is a repressor in fibroblasts and an activator in TG. The URI sequence was significantly activated by over-expressing the transcription factor CTCF in TG. (C) The UI construct is an activator in fibroblasts and a repressor in TG, while CTCF only activates in TG. Data shown are an average from at least two independent experiments performed in no less than four replicates. Error bars indicate S.E.M. One-way ANOVA indicates that the activating effect of CTCF is significant ^∗∗^*P* ⩽ 0.01, ^∗∗∗^*P* ⩽ 0.001*.*

**Fig. 3:**
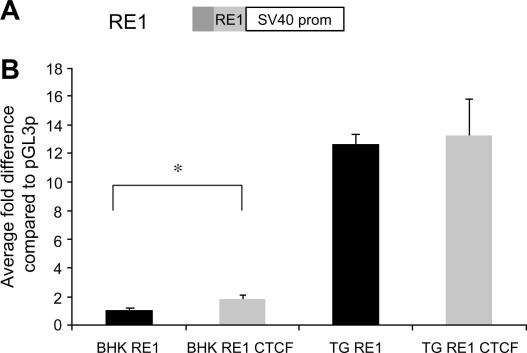
The RE1 demonstrates cell specificity; CTCF significantly activates transcriptional activity of the RE1 element in fibroblasts. (A) The structure of the RE1 insert in pGL3p. (B) The RE1 sequence was significantly activated by over-expressing the transcription factor CTCF in fibroblasts. Data shown from the RE1 alone are an average from at least two independent experiments performed in no less than four replicates. Error bars indicate S.E.M. One-way ANOVA indicates that the activating effect of CTCF is significant ^∗^*P* ⩽ 0.05.

In this communication we demonstrate using transient transfections that the URI domain, UI and RE1 components (Fig. 1) have different transcriptional activities in fibroblasts and TG (Figs. 2 and 3). The TG used were approximately 70% neuronal (Supplementary Fig. S1). Interestingly we saw that both UI and URI demonstrate opposite cell specific enhancer and repressor properties analogous to that of the RE1 in the context of pGL3p. PGL3p has a weak promoter supporting reporter gene expression and therefore the amount of reporter protein produced is dependent on the inserted regulatory domain. The URI locus can activate transcription in TG, whereas it strongly inhibits transcription in fibroblasts (Fig. 2).

The RE1 in the context of URI is a repressor in fibroblasts (Fig. 2B), as it can repress the activation mediated by the UI domain. Conversely, the RE1 acts as an enhancer in TG, both alone and when introduced into the TG specific repressor UI (Figs. 2B and 3). The RE1 is a cell specific enhancer as it supports a 12-fold increase in reporter gene expression in TG, whereas there was no significant change in fibroblasts (Fig. 3). Therefore, the RE1 has cell specific activity and can modulate the transcriptional activity of its flanking sequences.

**Fig. S2:**
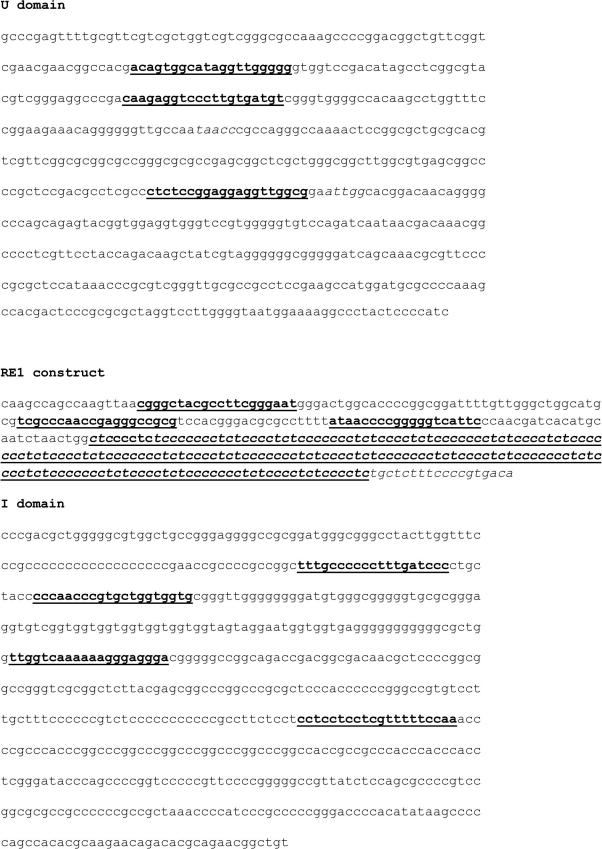
Potential CTCF binding sites CTCF consensus sequences are shown underlined, these sites were predicted using the CTCF binding site database and the published onsensus (Bao et al., 2008; Kim et al., 2007). There are 14 predicted sites in the RE1 construct, 7 predicted binding sites in the UI construct and 21 sites in the URI.

One of the cellular factors that could affect virus reactivation is the transcription factor CTCF, as it is responsive to cellular challenges including UV light [16,17] and is also a target for physiological stress [4], both of which can induce HSV-1 reactivation. Furthermore, it has been found that for some regulatory domains CTCF can act as either an activator or a repressor depending on the cell type [18]. CTCF activates the RE1 in fibroblasts but not in TG (Fig. 3), this could be due to maximal activation in TG prior to addition of CTCF. The larger UI domain contain 7 consensus sequences for CTCF (Supplementary Fig. S2), either in isolation or with the RE1 inserted (URI) is a regulatory domain that can enhance transcription in response to CTCF in TG (Fig. 2). This cell type specific response to CTCF, coupled with ∼15-fold relief of repression from the UI region in TG (Fig. 2C), demonstrates that the URI region may act as a neuron specific enhancer in response to CTCF. Therefore, changes in CTCF in response to stress may change the transcriptional activity of this region in neurons.

In genome wide analyses in Drosophila, CTCF is found to bind between closely spaced genes, which is an atypical distribution compared to other insulator proteins and suggest that it plays an active role in regulating gene activity [19]. CTCF binding regions are implicated in regulation of changes essential for the maintenance of latency and reactivation in HSV-1 [13,20,21]. An activity for CTCF at the UI and URI domains expands the repertoire of this transcription factor as a regulator of HSV-1 latency [21]. These data suggest the URI domain is an inducible, cell specific regulatory domain that could play a role in the regulation of the HSV-1 lifecycle.

**Fig. 4:**
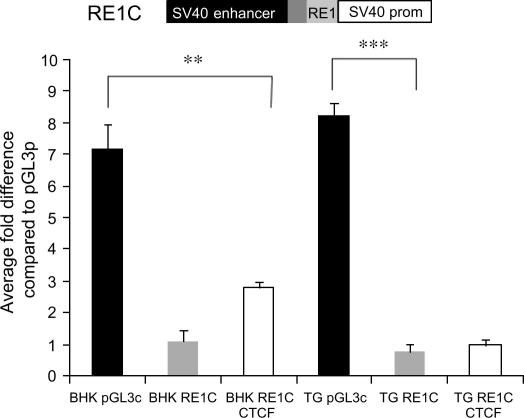
The RE1 can intercept the action of the SV40 enhancer and reduce transcriptional activity in fibroblasts and TG; CTCF can relieve repression exerted by RE1C in fibroblasts. (A) The RE1 is inserted between the SV40 enhancer and promoter in pGL3c. (B) Data shown are an average from at least two independent experiments performed in no less than triplicate. Two-way ANOVA indicates that the repressive effect of the RE1 is significant ^∗∗^*P* ⩽ 0.01, ^∗∗∗^*P* ⩽ 0.001.

In both fibroblasts and TG the RE1 domain acts as a repressor of an active enhancer when it was cloned into the pGL3c vector (Fig. 4B). The pGL3c construct utilises a strong enhancer to drive high levels of reporter gene expression in many cells. The levels of expression supported by the RE1C reporter construct are repressed to that supported by pGL3p in fibroblasts (Fig. 4B), whereas in TG the RE1C construct is repressed 15-fold relative to RE1 cloned into pGL3p. This suggests that the RE1 acts as an enhancer blocker in TG and this abrogates the mechanism by which the RE1 activates transcription. Further, transfection of CTCF into fibroblasts relieves repression of RE1C whereas in TG no effect is observed. This is analogous to the effect of CTCF on RE1 in pGL3p and suggests that CTCF has different functions on the RE1 in both cell types (Fig. 4B).

CTCF has been implicated as a major determinant controlling HSV-1 latency. A chromatin insulator-like element in the HSV-1 LAT intron, CTCF repeat long 2 (CTRL2), has homology to the RE1 and contains nine copies of the CTCCC CTCF consensus. CTRL2 has been shown to bind CTCF and demonstrated enhancer blocking activity in vitro [13], therefore it appears repeat sequences that can bind to CTCF may form a secondary structure which confers enhancer blocking activity. In Kaposi’s sarcoma herpes virus, CTCF binding regions prevent activation of nearby lytic genes [22]. This function is essential as it regulates spatial separation of permissive and non-permissive domains preventing inappropriate gene expression during latency and it may promote the co-ordinated cascade of gene expression during reactivation. Therefore, the RE1 in addition to many CTCF binding regions in HSV-1 may be important to prevent inappropriate activation of viral genes during latency and reactivation [13]. Although in transient transfections the DNA may or may not be complexed with histones 48 hours after transfection, in vivo the HSV-1 genome would be complexed with histones. The RE1 region has previously been identified as binding to nucleosome restructuring proteins, e.g. beta globin protein 1 (BGP-1) [23], therefore this region may be involved in remodelling nucleosomal structure, the co-ordination of which is crucial for the establishment of latency and reactivation of HSV-1 [20].

Taken together our data suggest this locus is involved in cell specific expression, and that the RE1 is key in the regulation of gene expression. Furthermore, the RE1 can act as an enhancer blocker, therefore it appears the locus may have different functions during the HSV-1 lifecycle. The data illustrates mechanisms by which tandem repeats can synergistically interact with their surrounding sequences to modulate enhancer function in a cell specific manner. An activity for CTCF at the UI and URI domains expands the repertoire of this transcription factor as a regulator of HSV-1 latency [21]. These data suggest the URI domain is a stimulus inducible, cell specific regulatory domain that could play a role in the regulation of the HSV-1 lifecycle. Indeed, we will pursue interactions between this locus and other transcription factors thought to be important in latency and reactivation of HSV-1.
